# Learning and achieving basic mental health competence in placement studies with the support of a tool: A qualitative study of student nurses’ experiences

**DOI:** 10.1016/j.ijnsa.2024.100219

**Published:** 2024-06-22

**Authors:** Siv Camilla Marriott, Ellen Karine Grov, Marianne Thorsen Gonzalez

**Affiliations:** a Faculty of Health and Social Sciences, Institute of Nursing and Health Sciences, University of South-East Norway, Drammen, Norway; b Faculty of Health and sport Sciences, Department of Health and Nursing Sciences, University of Agder, Kristiansand, Norway; c Faculty of Health Sciences, Department of Nursing and Health Promotion, Oslo Metropolitan University, Oslo, Norway

**Keywords:** Clinical competence, Mental health, Nursing education, Student placement

## Abstract

**Background:**

Learning basic mental health care competence is often challenging for the bachelor of science student nurses, and many lack basic mental health care competence to ensure safe and confident mental health care practice. Mental health assessment is an integrated part of this competence.

**Objective:**

The objective of this study was to explore and describe in depth how student nurses experience learning and achieving basic mental health competence while on mental health placement with the support of a learning tool.

**Design:**

An explorative and descriptive qualitative design was conducted to gain insight on how student nurses experienced learning basic mental health competence when on placement.

**Setting:**

A diversity of mental health placement settings in which student nurses were involved with patient care or welfare were approached; general psychiatric wards (*n* = 2), psychiatric ward for elderly people (*n* = 1), community mental health in-patient facilities (*n* = 2) and unconventional placements in the community (*n* = 9). Unconventional placements are a diversity of non-clinical service contexts.

**Participants:**

The participants comprised student nurses in their 3rd and final year while on mental health placement. Potential participants received information from course coordinators, the online learning platform, and from teachers in plenary. Using purposive sampling, 14 student nurses were recruited.

**Methods:**

Individual semi-structured interviews were conducted online and in person at two campuses of one university in Norway between August 2020 and December 2021. The interviews were transcribed and thematically analysed as described by Braun and Clarke.

**Results:**

Students expressed insecurity in a new clinical context. They engaged in new learning situations and realized the diversity of nursing practice. Unconventional placements were described as challenging contexts for learning basic mental health care competence.

**Conclusions:**

This qualitative study provided insight into how student nurses experience learning mental health assessment, and gaining relational, communicative, and ethical competence while on placement. Students revealed their insecurities and challenges in learning in a new context. Awareness of clinical learning opportunities on placement when preparing student nurses to learn basic mental health competence may help improve their confidence.

**What is already known**

Student nurses are anxious about their mental health placement.

Student nurses and newly qualified nurses do not feel competent in mental health care.

The quality of available placements in mental health care is diverse.

Alt-text: Unlabelled box

**What this paper adds**

Students on some unconventional placements struggle to gain basic mental health care competence as they have limited access to relevant learning situations.

Students expressed that they did not feel prepared for the mental health placement.

Students developed several relational and communicative competencies during their mental health placement.

Alt-text: Unlabelled box

## 1 Introduction

Concerns have been raised regarding nurses’ limited mental health care competence according to an integrative review by McInnes et al. (2022), and nurses report that the care they provide for people with mental health challenges, is of poorer quality than that for people with physical healthcare needs. Moreover, studies show that newly qualified nurses report lacking the necessary skills to confidently provide mental health care (Atashzadeh-Shoorideh et al., 2018; Hurley et al., 2020), and feel inadequately prepared to meet and care for people with mental health challenges (Liu et al., 2018; López-Entrambasaguas et al., 2019; McCann et al., 2009; McInnes et al., 2022). These studies are supported by studies reporting a lack of confidence or competence among nurses regarding their mental health education and practice (Crompton and Hardy, 2018).

From a clinical perspective, nurses are often in key positions and thus at frontline to recognize and respond to clinical signs of change or deterioration in mental health (Granek et al., 2019). Therefore, having the adequate experience and assessment competence, they might detect relevant changes. However, as nurses experience a lack of basic mental health care competence, their abilities to assess and identify deterioration in any healthcare setting are limited (Avery et al., 2020; Brunero et al., 2017; Sharrock et al., 2022). This can lead to unsatisfactory outcomes and put patients and service users at risk (Alexander et al., 2016). Thus, addressing how student nurses learn and achieve mental health assessment competence, is an important educational issue. This study addresses this concern. This corresponds well with the Norwegian Quality Improvement regulation – a national framework designed to ensure that both specialized and municipal healthcare providers establish a system for risk management and responsibility for internal control systems (Ministry of Health and Care Services, 2016). Additionally, the Norwegian national guidelines for nursing education require that institutions ensure that graduates hold the skills and knowledge needed to work professionally (Ministry of Education and Research, 2019).

## 2 Background

Clinical assessment, decision-making and critical thinking are vital parts of clinical competence, and thus important for student nurses to learn, develop and achieve during the educational process of becoming competent clinicians (Benner, 2008). Therefore, conveying how to recognize and identify core clinical signs of mental health issues is a central pedagogical approach.

Clinical signs of mental health issues are often characterized by significant disturbance in cognition, emotional regulation, or behavior (WHO, 2022). In their general (physical) nursing education, students are supported by various assessment tools, such as the Airway, Breathing, Circulation, Disability, Exposure (ABCDE) tool (Thim et al., 2012) or the National Early Warning Score (NEWS) (Royal College of Physicians of London, 2012). Such tools may support students in learning what to assess, how to judge and how to document accurately, but to date none have been developed for use within mental health. However, a quite recently published document analysis has identified a “key set” of clinical signs of mental health issues presented as a learning tool (Marriott et al., 2022). This tool was developed by reviewing and synthesizing international clinical mental health content including diagnostic manuals, nursing textbooks, screening tools and guidelines relevant for nursing and nursing education (Marriott et al., 2022).

From an educational perspective, the quality, extent, and educational level of mental health content in the bachelor of nursing education has been questioned in a comprehensive review of existing literature in the field (Barry and Ward, 2017), leaving findings of concern on mental health in the curriculum. Reported findings by Barry and Ward (2017) suggest that student nurses’ knowledge of mental health issues has been compromised due to variation and inconsistencies in the curriculum, the quality of theoretical and clinical learning opportunities, and student nurses’ level of knowledge was influenced by how they experienced the mental health content and placement.

Addressing how student nurses learn and achieve mental health assessment competence, is an important educational issue. Therefore, this study aimed to explore and describe how student nurses experienced learning and achieving basic mental health competence with the support of a mental health learning tool while on their mental health placement.

## 3 Method

### 3.1 Design

This study applied an explorative and descriptive qualitative design, aiming for an in-depth understanding of how students experience learning basic mental health competence while on placement.

### 3.2 Research context/setting

In Norway, the bachelor of nursing programme leads to authorization as a general nurse (RN). Currently, student nurses must complete an 8-week placement in a mental health setting, usually in their 3rd year of the bachelor of nursing programme.

Students’ mental health placement can be completed in a variety of clinical and non-clinical settings. The clinical settings include conventional mental health hospital wards, out-patient clinics, in-patient facilities in the community, various out-patient treatments such as FACT (Flexible Assertive Community Treatment) or ACT (Assertive Community Treatment). The non-clinical and often unconventional settings include a variety of charities, community services and other service providers offering low-threshold meeting places. Due to downsizing of mental health hospital wards globally, the availability of conventional mental health placements has been diminished (Andresen and Levin, 2014). Consequently, students are also offered unconventional placements, offering them an alternative experience to the treatment of acute mental illness (Allsop et al., 2013; Cowley et al., 2016).

While on placement, students will be allocated an RN as their supervisor. An academic nurse educator from the educational institution will monitor the students’ learning progression in close collaboration with the RN supervisor.

To support clinical learning of mental health assessment, students were equipped with the aforementioned assessment tool developed by Marriott et al. (2022), to which they were introduced and invited to try out during placement as part of their study participation. This tool addresses mental health assessment from both an observed and a subjectively expressed perspective. Main signs of clinical concerns in mental health care, such as risk concerns, psychotic concerns, substance use, and cognitive and self-care issues were briefly described. An elaborated overview of content included in this learning tool is outlined in Marriott et al. (2022).

### 3.3 Sample

Student nurses undertaking their mental health placement in the Bachelor of Nursing programme, were the study's target group.

Students were recruited from two different campuses of one Norwegian university. To recruit students, the head of the university department was formally contacted with regard to gaining access for student interviews. When approved, the same leader forwarded the information necessary to the mental health course coordinators requesting assistance with recruiting study participants. The course coordinators then posted study information and an invitation to participate on the digital learning platform Canvas. Authors one and three provided oral classroom information.

After the classroom presentation, students were handed written information about the study. Information was also sent by email, including about their right to withdraw at any point and instructions about contacting the first author directly for further information or to express interest in participation. Reminders were sent to the students twice by email. Those interested in participating in the study contacted the first author directly to schedule a time for interviews and filling out the consent form. During September 2020 and December 2021, three student groups were invited digitally and in classroom to participate (approximately 140 students). In total, fourteen student nurses at two university campuses were interested in participating. Of these, thirteen participants were female, and one was male, representing the gender distribution in education (Reform – Ressource Centre for Men, 2019). See Table 2 for an overview of the participants.

### 3.4 Data collection

A thematic interview guide informed by current literature (Braun and Clarke, 2006, 2019a, 2022) was developed. The interview guide included open questions, such as asking about students’ experiences of understanding the service users’ mental health challenges (the term 'service user', a term considered neutral and inclusive of all those receiving healthcare in this service, will be used in this document).

They were also asked to describe what they would do on a regular shift and reflect on what they had learnt on placement so far as well as their experience using the mental health assessment learning tool. During the interview, students were given the opportunity to add anything that was significant to them but had not come up as a topic or a question, at the end of the interview.

Individual interviews were conducted at the placement (*n* =  1), on campus (*n*  =  1), or via online video conferencing services such as Zoom or Teams (*n* = 12) and were audiotaped using the recording functions in these services. The interviews took place between September 2020 and December 2021. Due to the ongoing Covid pandemic, several institutions had restrictions on allowing anyone other than staff members into the facilities: hence the online solution. The interviews lasted 25–52 min and were audio-recorded and transcribed verbatim by a professional editing agency. The interviews were scheduled to take place within the last 3–4 weeks of the 8-week placement period to ensure that the students had sufficient experience to reflect on.

After the interviews, students were given formal written confirmation of their participation, stating that they had participated in a research study addressing student nurses’ basic mental health competence while on placement.

### 3.5 Data analysis

A reflexive thematic analysis encompassing the six phases outlined by Braun and Clarke (2006, 2019a, 2021, 2022) was used: 1) Familiarization with the dataset 2) Coding 3) Generating initial themes 4) Developing and reviewing themes 5) Refining, defining, and naming themes 6) Writing up. The analysis was led by the first author, with in-depth discussions aiming for consensus with the last author. Analysis was conducted from a pragmatic viewpoint, based on the philosophical assumption that knowledge is based on experience, and that each person's knowledge is unique as it is created by their experience (Kaushik and Walsh, 2019). Pragmatism is based on the epistemological assumption that research can avoid metaphysical arguments about the nature of reality and truth in favor of 'practical understandings' of specific, real-world problems (Patton, 2015). The emphasis of this perspective is on questioning the value and meaning of research findings through evaluation of its practical repercussions (Morgan, 2014).

After familiarization with the data, the interviews were coded on a semantic level (descriptive, inductive, participant-driven codes using raw data from the interviews). To reflect patterns in the data on a latent level (researcher-driven, interpreting underlying assumptions and concepts in the data), groups of codes were clustered into main themes and subthemes in line with the research question. To ensure credibility and dependability (Braun and Clarke, 2022), the first and the third author coded the interviews individually and thereafter discussed the codes. This process was repeated until a preliminary set of descriptive codes were generated to guide further analysis. The groups of codes helped identify patterns across the data, generating themes and subthemes. Both the first and third authors discussed and agreed on the final thematic outline. See Table 1 for an overview of themes and subthemes. Saturation was not utilized as a criterion to limit recruitment. Although Braun and Clarke, 2019b, Braun and Clarke, 2022 recommend avoiding using claims of saturation, we consider our data rich regarding the themes in this study. Rich data is characterized by the quality of data, including depth, diversity and complexity, rather than quantity (Fusch and Ness, 2015).

**Table 1:** Demographics of the student nurse participants.

Gender	Age	Participant	Former health care education	Former mental health work experience
F	21	11	No	No
F	25	4	No	No
F	23	7	No	No
F	22	8	No	No
F	22	6	Yes (2 years)	No
F	24	15	No	No
F	51	12	No	No
F	21	5	No	No
F	24	13	Yes (2 years)	No
F	52	10	Yes (2years)	No
F	22	9	No	No
F	22	14	No	No
F	24	3	No	No
M	23	2	No	Yes (2 years)

**Table 2:** Themes and subthemes describing experience learning and achieving mental health competence.

Themes	Subthemes
Experiencing mental health placement as a challenging learning context	Discovering new ways of nursing
	Feeling insecure in many clinical situations
	Having difficulties grasping nurses’ scope, role, and responsibility
Experiencing learning basic mental health care assessment supported by a tool	Struggling with identifying and differentiating the clinical signs available in the tool
	Reflecting on how assessment using a tool occurs in a variety of clinical situations
Experiencing learning basic mental health care competence in a nursing perspective	Gaining communicative and relational competence
	Acquiring ethical and personal competence
	Understanding how nursing assessment and mental health assessment are intertwined

The data analysis emphasized reflexivity throughout (Braun and Clarke, 2022; Dodgson, 2019). To enhance the quality of this study, several steps were undertaken as described by Braun and Clarke (2021), such as engaging with and identifying theoretical and philosophical assumptions; describing a variation of coding perspectives; flexibly interpreting the analytic process; actively and creatively conceptualizing themes; and reflecting on the authors’ own preconceptions throughout the analysis.

### 3.6 Ethical considerations

This research project was approved and registered by the Norwegian Agency for Shared Services in Education and Research (Sikt; project no. 947246). Participants were given written and oral information about the study and their right to withdraw at any time. All participants provided written consent. Data was stored in accordance with recommendations from the university. The first author was not professionally involved or associated with any parts of the undergraduate programme, and therefore unknown to the students. The third author had previously given a three-hour lecture on clinical assessment in mental health nursing to the classes approached.

## 4 Findings

Data analysis yielded three main themes: *Experiencing mental health placement as a challenging learning context; Experiencing learning basic mental health care assessment supported by a tool*; and *Experiencing learning basic mental health care competence in a nursing perspective.* Each main theme was characterized by two or three subthemes. An overview of the findings is presented in Table 1.

The findings suggest that learning in the mental health field was a new and different experience for many students. The findings are presented below, with illustrative quotes.

### 4.1 Experiencing mental health placement as a challenging learning context

The students experienced their mental health placement as a challenging learning context, deviating considerably from earlier placements. Consequently, they expressed feeling insecure in a variety of possible assessment situations. Moreover, they faced quite new ways of nursing, and thus struggled more with grasping the scope of nursing, and the roles, tasks, and responsibilities of nurses. The three subthemes characterizing this theme were; *Discovering new ways of nursing; Feeling insecure in many clinical situations*; and *Having difficulties grasping nurses’ scope, role and responsibility.*

#### 4.1.1 Discovering new ways of nursing

For many students, their mental health placement was their first encounter with a person facing mental health challenges and using mental health services. They discovered that nursing could mean simply being present and available for service users, and that collaborating with them in different activities was part of planned and organized therapeutic nursing practice.

*“We watch TV together, play Sudoku or quiz games, Yatzy and stuff like that. But on this ward, most patients are in an acute phase, so many are not capable of doing more than just being present.”* (P.13)

Students described gradually grasping that assessment situations were actually all these ordinary daily situations, and that they occurred during a car ride, or whilst visiting a farm or fixing a jigsaw together in the common area. Over time, they discovered and understood more of the complexity of the assessment situations in mental health care practice, albeit they did not quite understand how to use the tool. Students appreciated what they considered a quiet clinical context with more time to interact and collaborate with the service users.

*“It's just that this patient population is very different from what I have previously experienced. There are fewer practical tasks to do. Interpersonal relationships are highlighted here.”* (P.4)

However, discovering new ways of practice, also entailed facing feeling worried in this placement. Students revealed feeling unsafe or scared and felt that at times, some situations could be anxiety-provoking and dramatic, and that this was quite new for them:

*“And there was an incident, where this patient had left a letter and gone out, or in any case, locked their bathroom door. We thought maybe they were in there [the bathroom], so we had to unlock the door to check. It was a very emotional event; you know it was very uncomfortable. I remember having tunnel vision when we unlocked the door. And then they were not there, so there was a further search and alarming the police. But it was a very dramatic and very scary incident.”* (P.15)

Another experience students faced was service users expressing fear towards them as a student as the student might resemble someone who had caused the service user trauma. This experience of being confronted with a persons’ projections or negative transference was new. However, this experience helped the students to reflect on the importance of taking an insider perspective and always assess and be sensitive to a service users’ earlier emotional and relational experiences. Another experience shared by a student was about discovering, learning, and reflecting on the importance of balancing therapeutic closeness and distance, and how failing on this issue could activate service user's struggles with boundaries:

*“There was a patient that I had built rapport with, and we could talk, and he would share things with me. Eventually it became too much, and he started to become physically too close. That made me feel very insecure. In retrospect I have reflected on this, on what I did that contributed to this behavior. Did I send out any signals…? You reflect what you could have done differently, what happened and why it happened. After this incident I have been very careful in balancing closeness and distance*.” (P.14)

Many students also experienced that mental health practice entailed working independently and sometimes alone. Despite having had this experience in earlier placements, the observations, assessments, and situations they encountered when alone were new and unfamiliar to them. In these situations, students often felt stranded, in need of immediate supervision and support from more experienced professionals.

*“I discuss my observations with my supervisor. I still find it a bit difficult to respond to the service user, because I don't feel like there is much I can help them with yet. I still need reassurance from her – what she thinks or would suggest.”* (P.4)

Students often elaborated on their experiences differently, as the range of placements varied from charity cafes for people with substance/alcohol misuse issues to intensive psychiatric hospital wards. Many of the students on more unconventional placements considered that they would have learned more if they had a placement at an inpatient ward or outpatient clinic.

#### 4.1.2 Feeling insecure in many clinical situations

Students described themselves as quite insecure about how to understand and make sense of these signs, despite having the tool. Likewise, not understanding why a change in mood or affect had appeared, could make them feel quite puzzled. They described feeling anxious about doing or saying something wrong in assessment situations, considering that this might trigger the service user – something quite vividly expressed by one student:

*“I have reflected, that when I have heard statements like «I don't want to live anymore» - it's difficult because what are you supposed to say? You want to give a correct response… So, when people have made such comments, I have tried to shift focus to them and likely asked if this is something they have thought about for a long time or why are you thinking about that*?” (P.9)

Students reflected on their role as a student and argued that this position could make it difficult to act appropriately, particularly when experiencing someone expressing suicidal thoughts:

*“I cared for a service user who started talking about … They were in a life-crisis and talked about committing suicide and presented plans on how to do so. I was alone with the service user at the time, so I didn't have anyone to lean on. That made me insecure. How should I handle this? It worked out ok because there was nothing I could say or do to help. They just needed someone to talk to*.” (P.14)

#### 4.1.3 Having difficulties grasping nurses’ scope, role, and responsibility

Students experienced struggling to grasp the scope of nursing in the context of mental health, and felt that understanding the nurse's role and responsibilities was unclear:

*“Basically, everyone does everything. The only thing for which the nurses have extra responsibility is related to medication management. Distribution of medication and things like that. Apart from that, everyone does everything*.” (P. 13)

Consequently, they also struggled with what was expected of them as student nurses and highlighted that they understood that nurses had particular responsibility for administrating medication as well as “keeping an eye on” the service users’ self-care needs. Despite the tool explicitly having a nursing scope for assessment, they considered that these were the two issues distinguishing nurses’ scope, role, and responsibility from the other professions in this multi-professional context. Moreover, they often came up with nursing scope assessments related to more physical health issues, like physical symptoms or wounds:

*“That's when the nursing role becomes very clear. That you have an overview of physical health needs and physical symptoms*.” (P.2)

Other students missed the busy general wards – with their clear expectations of competence levels and having concrete tasks to do, like medical/nursing procedures or using different validated physical screening tools – and it was often unclear to them, what they were expected to do, assess, and follow up:

*“I find it very difficult… I feel that on my placement there is not so much focus on specific tasks. It's more like «how was your day? ». Symptoms or signs of disorders are not highlighted… It is more about how the day has been*.” (P.4)

### 4.2 Experiencing learning basic mental health care assessment supported by a tool

Students struggled with what to observe, and how to find meaning in what they observed. Two subthemes characterized this theme: *Struggling with identifying and differentiating clinical signs available in the tool*; and *Reflecting on how assessment using a tool occurs in a variety of clinical situations.*

#### 4.2.1 Struggling with identifying and differentiating clinical signs available in the tool

Students revealed that they had been involved in learning situations entailing the possibility to observe and identify a diversity of clinical signs associated with mental health challenges. They mentioned observing psychotic symptoms, low mood, anxiety, inappropriate behavior or communication, and unmet self-care needs due to mental health challenges, all signs in line with the provided learning tool. Depending on the nature of the placement, students also described having observed clinical signs associated with eating disorders, cognitive impairment, substance use and relational issues. However, identifying, differentiating, and making sense of these signs was not easy:

*“In my experience, I find it difficult to separate signs of substance use from signs of mental health disorder if I meet someone who is under the influence. I can't pinpoint whether the person was psychotic or high*.” (P.13)

Students further articulated that it could be difficult to differentiate what they had experienced, as they realized that many clinical signs appeared across diagnoses, often named ‘transdiagnostic’. Moreover, students reflected that learning would take time and lots of experience.

Students who experienced being on placements where assessment, healthcare and documentation were relevant learning situations, reflected that having a tool available was useful, and said that they had used it as a reference guide for confirming their own observations.

*“I have had a look at it [the learning tool], and I found it instructive. Some of the points/content are things that I have not encountered, like psychosis. But I have got an idea about what to look for, even if I haven't experienced it. And I have seen that some of the other points match what I have observed. So, in a way I get a confirmation that my observations were correct.” (P.8)*

However, students on some of the more unconventional placements found the tool neither suitable nor applicable for their context, and therefore they had only briefly used it. At these unconventional placements, availability of sufficient background information about the service users made it even more challenging to understand what would be relevant to assess:

*“I found it a bit difficult at the community mental health meeting-place, experiencing mental health challenges. Maybe it is too challenging for me when it is not obvious? When I have no previous experience, it becomes unclear to me.”* (P.7)

#### 4.2.2 Reflecting on how assessment using a tool occurs in a variety of clinical situations

Students gradually grasped assessing from a nursing perspective and revealed concerns about the importance of nurses taking responsibility for assessing basic self-care needs. Both the more physical aspects and psycho-social self-care needs were mentioned:

*“You look at their appearance, routines, nutrition, and the social needs. Many people withdraw, are not groomed, or haven't showered or changed clothes for many days*.” (P.14)

They addressed psychosocial needs by facilitating a safe environment and engaging, thereby building trust and relationships with the person struggling with mental health challenges. They also expressed how they had striven to assess how the service users experienced their situation from a person-centered or inside perspective. However, in these more therapeutic learning situations, they were often confronted with a lack of therapeutic experiences and competence:

*“I try to ask about how he or she is feeling, but I find it difficult. I feel that it is hard to know what to say when you are inexperienced*.” (P.3)

Students appreciated learning situations involving assessment when following up service users over some time. These situations provided learning opportunities involving observing changes related to mental health state over time, especially when assessments involved outcomes or the effects of pharmaceuticals or therapeutic strategies:

*“In the case of notice, the most change [in patients’ mood or behavior] is when they have started on a new medication or change of dosage. That is probably where the change is most noticeable if you don't know the patient*.” (P.10)

On unconventional placements, students found that staff did not have a mental health assessment focus. Students clearly expressed disappointment and criticism towards this kind of placement. Moreover, they struggled with using factual learning situations, as their placement deviated from those presented in their curriculum.

*“I will give feedback to the university, having the placement at the community mental health meeting-place was not beneficial for my learning. It's all about drinking coffee and chatting, and that's nice, but I think I need to experience what's described in the textbooks and learning tool in practice. To experience it for myself because that makes it easier to connect theory and practice*.” (P.7)

### 4.3 Experiencing learning basic mental health care competence in a nursing perspective

Students realized that their new learning experiences had expanded their understanding of mental health challenges, and thus contributed to their mental health care competence. The three subthemes characterizing this theme were: *Gaining communicative and relational competence; Acquiring ethical and personal competence*; and *Understanding how general nursing assessment and mental health assessment are intertwined.*

#### 4.3.1 Gaining communicative and relational competence

Students were particularly engaged when talking about their experiences of learning and gaining communicative and relational competence and reflected upon experiencing and learning a variety of therapeutic approaches applicable for future placements and their nursing career. They highlighted learning about the importance of developing trust, building relationships and how to address vulnerable issues in a therapeutic way:

*“If you get to know them, it becomes easier to understand which questions you can ask. I'm a bit anxious about… Early on I was insecure about what would be ok to address [with patients] Will they get upset with me? But you quickly realize what you can address and not. And they are actually quite open*.” (P.11)

The students had experienced success in building trust and engaging in relational processes, as well as developing clinical competence:

*“I have recently gained the confidence of a woman who has been very open and shared her experience of her illness. I have found it difficult to deal with this information, on the other hand it is nice to gain that trust.”* (P.15)

Students highlighted the importance of establishing a person-cantered attitude and being respectful. They revealed in detail how they had applied therapeutic assessment strategies such as active and empathic listening and different affirmative communication styles, something that could be quite demanding and challenging.

*“There are two people with borderline personality disorder on placement, so the mood is fluctuating. You become insecure and very careful about what you are saying. Because suddenly what you say can trigger a lot of anger or sadness. That has been challenging for me to face, especially since I am a person who talks a lot… Well, I have become much better at weighing my words and not respond so quickly. I have become very aware of the significance of communication*.” (P.15)

Students clearly understood the importance of just being present, available, and often silent, rather than being talkative and offering advice, as one student reflected:

*“Yesterday there was this one [patient], he walked and walked, had a cigarette, and continued walking… But then he sat down next to me. I thought that I would acknowledge that he was sitting there with me. Furthermore, I needed to adjust my behavior towards him. It would be natural to start a conversation if you want to approach another person. Maybe ration the questions out a bit … Be gentle*.” (P.10)

Students were consciously aware of service users struggling with trust issues from the past and realized that a placement of only a few weeks could be too short for establishing trustful relations. However, they pointed out that having a relationship with the service user was vital when attempting to collaborate or to establish a dialogue on important issues related to their situation.

*“They [patients] often have many challenges related to childhood and upbringing, which can make it difficult for them to gain trust with other people. It is not so easy to come as a student when they know that I will leave after 8 weeks.”* (P.15)

Students recognized that by participating in everyday activities, such as going for a walk or playing a board game, provided opportunities to get to know the person and gain their trust.

#### 4.3.2 Acquiring ethical and personal competence

Students were alerted to several ethical issues in their mental health placement and elaborated on how their own prejudices and stereotypes regarding people with mental health challenges had been positively corrected in this placement. This was particularly related to prejudices that “all patients with mental health disorders are dangerous":

*“Because this has been a population that I have been a bit afraid of, but when I realized that they are scared because they have scary experiences [perceptions] it makes it easier to understand. At the same time also incomprehensible*.” (P.13)

Encountering with persons experiencing mental health challenges had a deep emotional impact on the students, and they revealed how that had contributed to developing their own empathic capacity, thereby increasing their personal competence. Moreover, they were emotionally touched by service users’ stories and lived experiences. They expressed empathy and compassion with the service users’ experiences of suffering and were genuinely happy when things worked out well for them.

*“I would also be scared if I felt someone was stalking me or was going to poison me. You know, it is natural to be scared and angry about that. But I can't really imagine what it is like to feel that someone is going to harm you all the time*.” (P.13)

One student also experienced how a service user had shown concern, guilt and a capacity for empathy for her as a student, when she shared something really painful from her life:

*“A patient shared something that was painful, and then apologized for doing that. It was very difficult to listen to. Considering that I am quite young and inexperienced, the patient probably thought it could be hard for me to deal with*.” (P.14)

#### 4.3.3 Understanding how nursing assessment and mental health assessment are intertwined

Students revealed that during their placement they had increased their understanding of mental health care assessment, and managed to grasp how nursing assessment and mental health assessment were connected. Moreover, they gained an understanding of how the nursing experiences and knowledge they had prior to this placement gradually also became relevant in mental health care. They realized that they had applicable skills and developed an understanding of combining current competence with this new learning experience.

«*Sleep for instance, is very difficult for a lot of people, and may contribute to the admission. They lack sleep. That's one of the most important things to address on the ward; making sure that they get to sleep, eat, drink, and feel safe enough to relax a bit. I have witnessed that after one night with proper sleep, someone who was psychotic the previous night is doing much better the next day”* (P.13)

Students were also able to reflect on how people's emotional or mental health state might influence their living conditions and personal dignity.

*«I went to see her [the patient]. She told me that she didn't see any value in being alive anymore, that she was not well at all. That was painful to hear. And maybe the way her home looked reflected how she felt. A lot of food and rubbish … dirty clothes everywhere»* (P.9)

In addition to merging previous knowledge and experience with their mental health care experience, students also indicated that what they had learned about mental health care were transferable skills, valuable in any future healthcare setting they would practice.

## 5 Discussion

The aim of this study was to explore and describe how student nurses experienced learning and achieving basic mental health competence while on placement, with the support of a learning tool. The findings revealed that student nurses found the new context challenging and that understanding the scope of nursing in mental health care was difficult. Students on unconventional placements struggled to identify signs of mental health challenges despite having access to a learning tool. They suggested that this was due to the lack of relevant learning situations.

### 5.1 Mental health placement as a challenging learning context

Student nurses experienced some challenges in the context of mental health care learning and felt inadequately prepared. They further revealed facing unfamiliar nursing practices and learning situations, and thus needing support in identifying and engaging in these situations. This might have negatively influenced their learning, as students link knowledge most effectively when they achieve understanding through interaction and varied approaches (Kober et al., 2015). Moreover, as novice learners, students often possess less developed or incomplete conceptual frameworks that can guide them in unfamiliar settings (Kober et al., 2015), which might also explain why students felt stressed and unprepared.

Students learn by building on previous knowledge when trying to understand new content (Dong et al., 2020). Several learning theories and perspectives address how students learn and develop competence, wherein situated learning theory (SLT), applying a sociocultural learning theory, developed by Lave & Wenger (1991), is relevant. SLT is characterized by the assumption that skills must be learned in the authentic situated social context that provides the relevant activities, and thus placement is the core learning arena. Within the complexity of a placement arena, students are naturally involved in learning activities entailing critical thinking and recalling relevant knowledge (Lave and Wenger, 1991). Students struggled to learn mental health assessment. As some placements were unconventional and so did not provide relevant learning activities, this might explain both the students’ struggle with learning mental health assessment and the possibilities for their using this learning tool as a pedagogical support.

Our findings are in line with the learning perspective of Vygotsky et al. (1978), which suggests that learners develop a higher cognitive level of understanding when they are supported by more experienced supervisors or in close collaboration with more knowledgeable peers. Students identified some learning gaps when they sometimes felt lost and in need of supervision in unfamiliar situations. On the other hand, students elaborated on and understood what they had gained while on placement. Lave and Wenger (1991) stress the significance of connections and exchanges between beginners and more knowledgeable persons (in this context clinicians), a perspective further supported by the ideas of Vygotsky et al. (1978). On this issue, students felt that, despite having access to experienced RN supervisors and a learning tool, these resources did not make up for the lack of relevant learning activities when learning mental health assessment. Simulating using the learning tool prior to placement might have given students more confidence in using it.

Students in mental health placements often expressed insecurity due to unstable service users, leading to elevated stress levels due to potential unpredictable behavior (Al-Zayyat and Al-Gamal, 2014; Wojtowicz et al., 2014). Students often remained passive in clinical situations, worried about triggering service users’ behavior. The learning tool might have helped guide students in identifying clinical signs. Clinical placements in mental health is different from a more task-oriented practice (Acı et al., 2022), as skills in handling cognitive, emotional, and behavioral issues are required. It is, however, reported that students who have been successful at establishing meaningful relationships with service users on placement experienced higher levels of clinical confidence (Demir and Ercan, 2018; Zhang et al., 2021). This underlines the importance of students engaging with service users.

Many students questioned the learning possibilities in the mental health placement, and assessment was quite often not an issue. This obviously hindered learning assessment of mental health clinical signs and assessment in a nursing perspective. These learning realities are a worldwide challenge, and might contribute to newly educated nurses reporting marginal mental health competence (McInnes et al., 2022). In contrast to the above arguments, research suggests that student nurses are content with their mental health placement, regardless of whether it is conventional or unconventional (Bjørk et al., 2014; Cowley et al., 2016; Missouridou et al., 2021). One possible explanation could be that students feel welcome and included at the placement. They may have a good relationship with their RN supervisor, but the factual learning can still be limited.

### 5.2 Learning basic mental health care competence despite challenges

Despite facing challenges, over time students experienced becoming less task-oriented and were able to put words to and elaborate on experiences in which they had been engaging. They further realized how engaging in activities together with the service users gave them useful information about the persons’ struggle and emotional state. Some of these meetings also allowed the service user to trust them enough to reveal their subjective lived experiences. To some degree, they increasingly grasped the nurses’ scope and role. These findings are interesting in the context of the findings reported by Molloy et al. (2020), who found that students experienced an enhanced understanding of their role possibilities when engaging with service users in a therapeutic recreational program.

From a theoretical perspective, the abovementioned findings fit well with the theoretical perspective of Vygotsky et al. (1978) and the sociocultural learning perspective of Lave & Wenger (1991). From a research perspective, they concur with research suggesting that students’ stress decreased when direct clinical experience in placement increased (Al-Zayyat and Al-Gamal, 2014) and with longer exposure to mental health care practice (Thongpriwan et al., 2015).

The findings revealed that the students felt unprepared, mirroring recent findings (Marriott et al., 2023) on how, despite nurse educators creatively and wholeheartedly applying a variety of pedagogical approaches to optimally prepare students, they still struggled to grasp core aspects of what to learn. These nurse educators were constantly aware of the students’ struggles and the challenges of the marginal mental health learning contexts. Therefore, they engaged in students being theoretically and personally prepared as well as supporting them in integrating theory and practice (Marriott et al., 2023). Being prepared and well informed is reported to positively influence students’ learning (Keeping-Burke et al., 2020), and is therefore a pedagogical issue. However, students’ background, experience and expectations will always vary, as will the placement's learning context and ward atmosphere, and it might seem unrealistic that students will ever feel fully prepared for their mental health placement.

However, when students gradually became more comfortable and confident, and developed meaningful relationships with service users, they gradually understood more and could take advantage of the existing learning situations, recognizing playing board games, fixing jigsaws, and going for walks or a drive with service users as meaningful activities relevant to nursing assessment and interventions. This competence development can be understood in light of Lave and Wenger (1991) perspective of legitimate peripheral participation (LPP) on learning, viewing learning as something embedded in everyday activity, context and culture, and often unintentional. As the students became more experienced and confident on their placement, it became clear that they integrated and built on their previous knowledge and clinical experience. This is a key concept in learning (Kober et al., 2015; Vygotsky et al., 1978). Moreover, students realized that there was a link between lack of sleep, poor nutrition, reduced self-care capacity and an increase in clinical signs of mental health issues and challenges. This development is supported by Lave and Wenger (1991) learning perspective implying that when students become more experienced and knowledgeable, they make assumptions, take on more responsibility and become a more central member of the practice community.

Both healthcare professionals and students training in healthcare professions are confronted with a variety of ethical issues in their clinical practice. Students seemed to grasp that having mental health problems caused different behavior and challenges. Consequently, they developed an understanding highlighting ethical reflections and why they needed to use a different approach when facing difficult situations. An integrative review confirms that students face ethical challenges in daily care and treatment (Lechasseur et al., 2018). This requires addressing through ethical sensitivity, knowledge, reflection, behavior, and decision-making. Students in this study also recognized these issues.

Students further revealed an empathic and generally positive attitude towards the people they were caring for, with some exceptions related to experiences from intimidating situations. This finding is in accordance with similar findings recently reported by Alexander et al. (2023).

### 5.3 Strengths and limitations

The strengths of this study are that the findings are developed from students’ recent experiences and reflections as they were interviewed while on placement. Another strength is that students were provided an assessment learning tool, as to our knowledge has not been explored earlier. This study is further strengthened by a sample of 14 student nurses across a diversity of placement settings. A study limitation could be that the students were from the same university.

### 5.4 Conclusion

The findings in this study revealed that student nurses experienced mental health placement as a challenging learning context, and that they struggled with identifying and assessing clinical mental health signs. Despite having a comprehensive learning tool at their disposal, this tool had limited utility due to their scarce and often unconventional mental health placements. The findings highlight the need for improved learning possibilities in mental health placements that offer possibilities where students can practice mental health assessment.

### 5.5 Recommendations for clinical practice

Based on the findings, it is recommended to facilitate realistic learning situations either in simulation training or placement and strengthen mentoring and support during placement. It is also recommended to involve the learning tool in simulation training before placement. On an institutional level, it is recommended to strengthen the collaboration between placement and the educational institutions on course curricula and learning outcomes

### 5.6 Recommendations for further research

Based on our findings, further research should explore a variety of facilitated and arranged mental health learning situations and how students can optimally benefit from these. Furthermore, studies investigating how students can learn and achieve competence in simulating training by using a mental health assessment learning tool.

## Funding

The research received no specific grant from any funding agency in the public, commercial or non-profit sectors.

## Ethical approval

This research project was approved and registered by the Norwegian Agency for Shared Services in Education and Research (Sikt; project no. 947246).

## Data availability

Due to the small sample size, our data are not suitable for sharing as participants could be recognized.

## CRediT authorship contribution statement

**Siv Camilla Marriott:** Writing – review & editing, Writing – original draft, Validation, Project administration, Methodology, Formal analysis, Conceptualization. **Ellen Karine Grov:** Writing – review & editing, Writing – original draft, Validation, Supervision, Methodology, Formal analysis, Conceptualization. **Marianne Thorsen Gonzalez:** Writing – review & editing, Writing – original draft, Validation, Supervision, Project administration, Methodology, Formal analysis, Conceptualization.

## Declaration of competing interest

The authors report no conflict of interest, as the authors alone are responsible for the content and writing of this paper.

## Appendix Supplementary materials

Image, application 1

Image, application 2

Image, application 3
