# Supplementary material for: Learning and achieving basic mental health competence in placement studies with the support of a tool: A qualitative study of student nurses’ experiences
Source: Int J Nurs Stud Adv. 2024 Jun 22;7:100219. doi: 10.1016/j.ijnsa.2024.100219 (PMC11278879; doi:10.1016/j.ijnsa.2024.100219)
Supplement: Supplementary file 2 [file mmc2.docx]

Data availability

Due to the small sample size, our data are not suitable for sharing as participants could be recognized.
