# Supplementary material for: Learning and achieving basic mental health competence in placement studies with the support of a tool: A qualitative study of student nurses’ experiences
Source: Int J Nurs Stud Adv. 2024 Jun 22;7:100219. doi: 10.1016/j.ijnsa.2024.100219 (PMC11278879; doi:10.1016/j.ijnsa.2024.100219)
Supplement: Supplementary file 3 [file mmc3.docx]

Table 1. Demographics of the student nurse participants

| Gender | Age | Participant | Former health care education | Former mental health work experience |
| --- | --- | --- | --- | --- |
| F | 21 | 11 | No | No |
| F | 25 | 4 | No | No |
| F | 23 | 7 | No | No |
| F | 22 | 8 | No | No |
| F | 22 | 6 | Yes (2 years) | No |
| F | 24 | 15 | No | No |
| F | 51 | 12 | No | No |
| F | 21 | 5 | No | No |
| F | 24 | 13 | Yes (2 years) | No |
| F | 52 | 10 | Yes (2years) | No |
| F | 22 | 9 | No | No |
| F | 22 | 14 | No | No |
| F | 24 | 3 | No | No |
| M | 23 | 2 | No | Yes (2 years) |
